# Generation of a new mouse model of glaucoma characterized by reduced expression of the AP-2β and AP-2δ proteins

**DOI:** 10.1038/s41598-017-11752-6

**Published:** 2017-09-11

**Authors:** Maria Monica Barzago, Mami Kurosaki, Maddalena Fratelli, Marco Bolis, Chiara Giudice, Laura Nordio, Elisa Cerri, Luciano Domenici, Mineko Terao, Enrico Garattini

**Affiliations:** 1 Laboratory of Molecular Biology, IRCCS-Istituto di Ricerche Farmacologiche “Mario Negri”, via La Masa 19, 20156 Milano, Italy; 2 DIVET, Faculty of Veterinary Medicine, University of Milan, Italy, Via Celoria 10, 20113 Milano, Italy; 3 Consiglio Nazionale delle Ricerche (CNR), Neuroscience Institute, Pisa, Italy; 4 Department of Applied Clinical Sciences and Biotechnology (DISCAB), University of L’Aquila, L’Aquila, Italy

## Abstract

We generated 6 transgenic lines with insertion of an expression plasmid for the R883/M xanthine dehydrogenase (XDH) mutant protein. Approximately 20% of the animals deriving from one of the transgenic lines show ocular abnormalities and an increase in intra-ocular pressure which are consistent with glaucoma. The observed pathologic phenotype is not due to expression of the transgene, but rather the consequence of the transgene insertion site, which has been defined by genome sequencing. The insertion site maps to chromosome 1qA3 in close proximity to the loci encoding AP-2β and AP-2δ, two proteins expressed in the eye. The insertion leads to a reduction in AP-2β and AP-2δ levels. Down-regulation of AP-2β expression is likely to be responsible for the pathologic phenotype, as conditional deletion of the *Tfap2b* gene in the neural crest has recently been shown to cause defective development of the eye anterior segment and early-onset glaucoma. In these conditional knock-out and our transgenic mice, the morphological/histological features of the glaucomatous pathology are surprisingly similar. Our transgenic mouse represents a model of angle-closure glaucoma and a useful tool for the study of the pathogenesis and the development of innovative therapeutic strategies.

## Introduction

Glaucoma is one of the leading causes of blindness and it is characterized by a global prevalence of 3.5% in the population aged 40–80 years^1^. Glaucoma is a multifactorial and heterogeneous disease which eventually leads to visual deficits^2^. Increased intraocular pressure is a major risk factor for glaucoma and it is the only clinically treatable feature. Primary open-angle glaucoma is the most prevalent form, although angle-closure glaucoma is also widespread, particularly in individuals of Asian descent^3^.

Angle-closure glaucoma is predominantly the result of congenital disorders which cause a defective development of the anterior segment of the eye, also known as anterior segment dysgenesis (ASD)^4^. The anterior segment of the eye is a very complex system and includes the cornea, iris, lens, ciliary body and various optic fluid drainage structures, such as the Schlemm’s canal. From a developmental point of view, the anterior segment originates from the interactions among the surface ectoderm, the optic cup, the mesoderm and the neural crest. Deficits in the development of the anterior segment cause structural and functional abnormalities in the system responsible for the production and drainage of the aqueous humor. In particular, defective reabsorption of the eye fluid results in increased intra-ocular pressure (IOP) which is a major predisposing factor for the development of glaucoma^5–7^. There are a number of genes whose mutations have been associated with human ASD^4, 8, 9^. However, it is likely that other as yet unidentified genes play a role in the genesis and development of ASD and subsequent angle-closure glaucoma. For instance, there is evidence that alterations in the regulation/activity of transcription factors belonging to the AP-2 family are involved in the generation of glaucoma^10^. This family consists of five members, AP-2α, AP-2β, AP-2γ, AP-2δ and AP-2ε. AP-2β is believed to play a crucial role in the development and function of the neural crest^11^. Indeed, removal of AP-2β, the product of the *Tfap2b* gene, from the mouse neural crest and derivatives by means of the Cre-Lox technology, was recently shown to result in ASD and early-onset glaucoma^10^.

Xanthine dehydrogenase (XDH)^12–16^ and aldehyde oxidases (AOXs) are molybdo-flavoenzymes characterized by remarkable structural similarity^17, 18^. Unlike AOXs which are endowed with broad substrate specificities, XDH specifically oxidizes hypoxanthine into xanthine and xanthine into uric acid^19, 20^. Given this substrate specificity, XDH is considered to be the key enzyme in the catabolism of purines. The catalytically active XDH protein is a homodimer consisting of two identical subunits, which are the product of the corresponding gene. During the course of structural and functional studies on mouse XDH, we generated an XDH protein (*Mut*-*XDH*) which contains an amino acid substitution (R883/M). The substitution reduces the ability of XDH to recognize hypoxanthine and xanthine as substrates^21^. In addition, *Mut*-*XDH* exerts a dominant negative effect *in vitro*, as it maintains its ability to dimerize with the wild-type subunit. To evaluate the potential of the mutant protein to act as a dominant-negative factor and to specifically silence the native enzyme *in vivo*, we generated *Mut*-*XDH* transgenic mice.

The present study reports on the characterization of a *Mut*-*XDH* transgenic line of mice which, unexpectedly, is characterized by increased intra-ocular pressure (IOP) and post-natal development of ocular abnormalities consistent with angle-closure glaucoma. Increased IOP and angle-closure glaucoma are observed only in mice homozygous for the transgene. Overall our data support the idea that the glaucomatous phenotype is not caused by expression of *Mut*-*XDH*, but it is rather due to the serendipitous transgene insertion into a small region of chromosome 1 in close proximity to the *Tfap2b* gene coding for AP-2β. As a consequence of this insertion, AP-2β expression is dramatically reduced in transgenic mice with the glaucomatous phenotype. Interestingly, the macroscopic and microscopic characteristics of the pathologic eyes of our homozygous transgenic animals and of the mice with conditional deletion of *Tfap2b* are remarkably similar^10^. Taken together, the evidence gathered from the characterization of our transgenic animal provides independent support to the idea that deficits in the expression of AP-2β cause angle-closure glaucoma in mice. In addition, our transgenic mouse represents an important addition to the few animal models of glaucoma available^22–25^. In general terms, our study underlines the importance of defining the insertion site of a transgene into the mouse genome, as phenotypic traits observed in genetically engineered mice may not be always attributable to a direct action of the transgene itself.

## Results

### Generation of a transgenic line of mice characterized by a pathologic phenotype consistent with glaucoma

**Figure 1:**
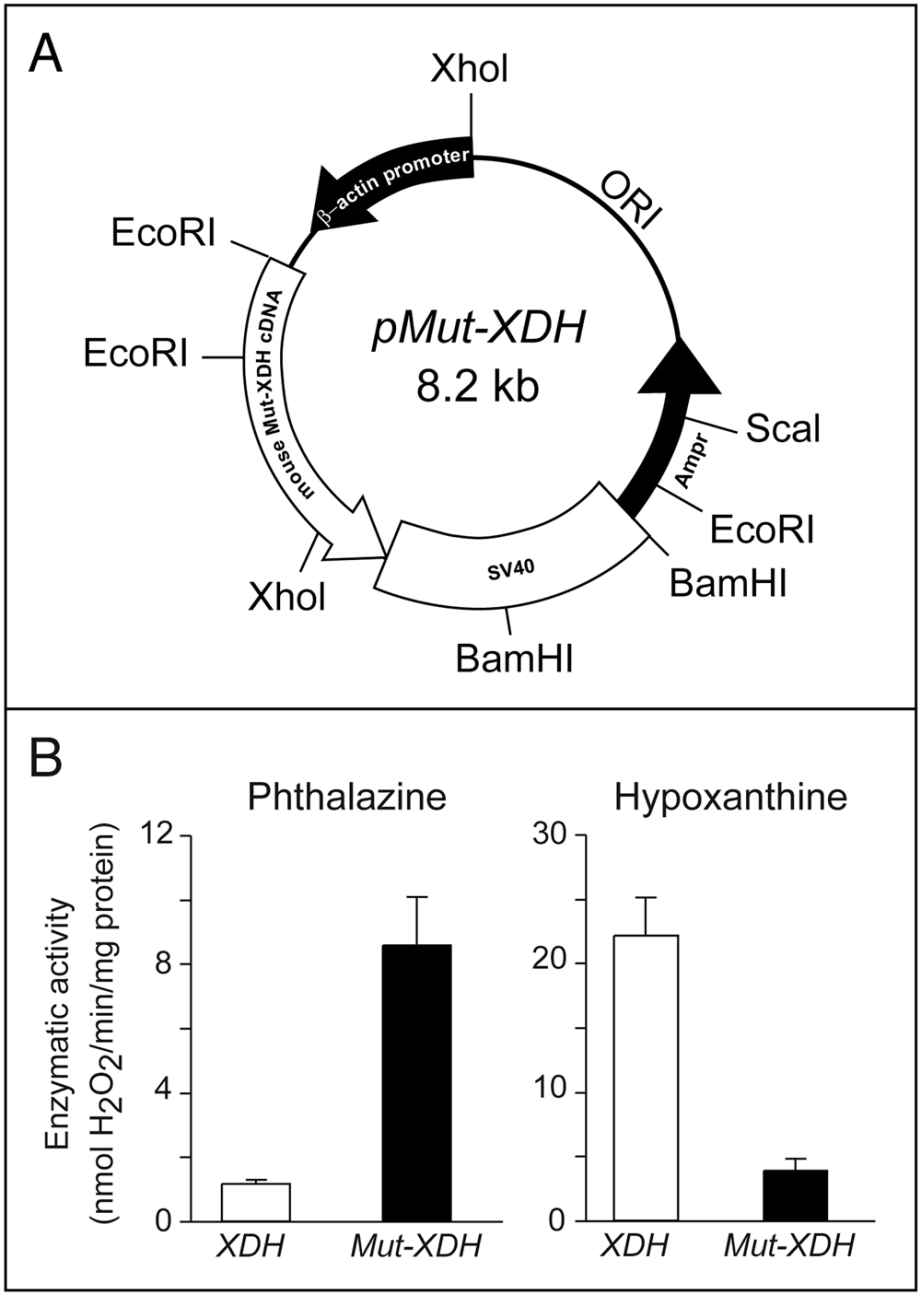
Structure of the plasmid used for the generation of transgenic mice and enzymatic activity of the XDH and MUT-XDH proteins. (**A**) The panel illustrates the structure of the expression plasmid containing the cDNA coding for the R883/M mutant XDH protein which was used for the generation of the transgenic mice. (**B**) Parental (*XDH*) and R883/M XDH mutant (*Mut*-*XDH*) proteins were expressed in HEK-293 cells stably transfected with the corresponding expression plasmids. The same amount of semi-purified protein extracts were used to determine XDH enzymatic activity using phthalazine and hypoxanthine as substrates. The amount of H_2_O_2_ produced during the enzymatic reaction was measured. Each value is the mean ± SD of three replicates.

**Figure 2:**
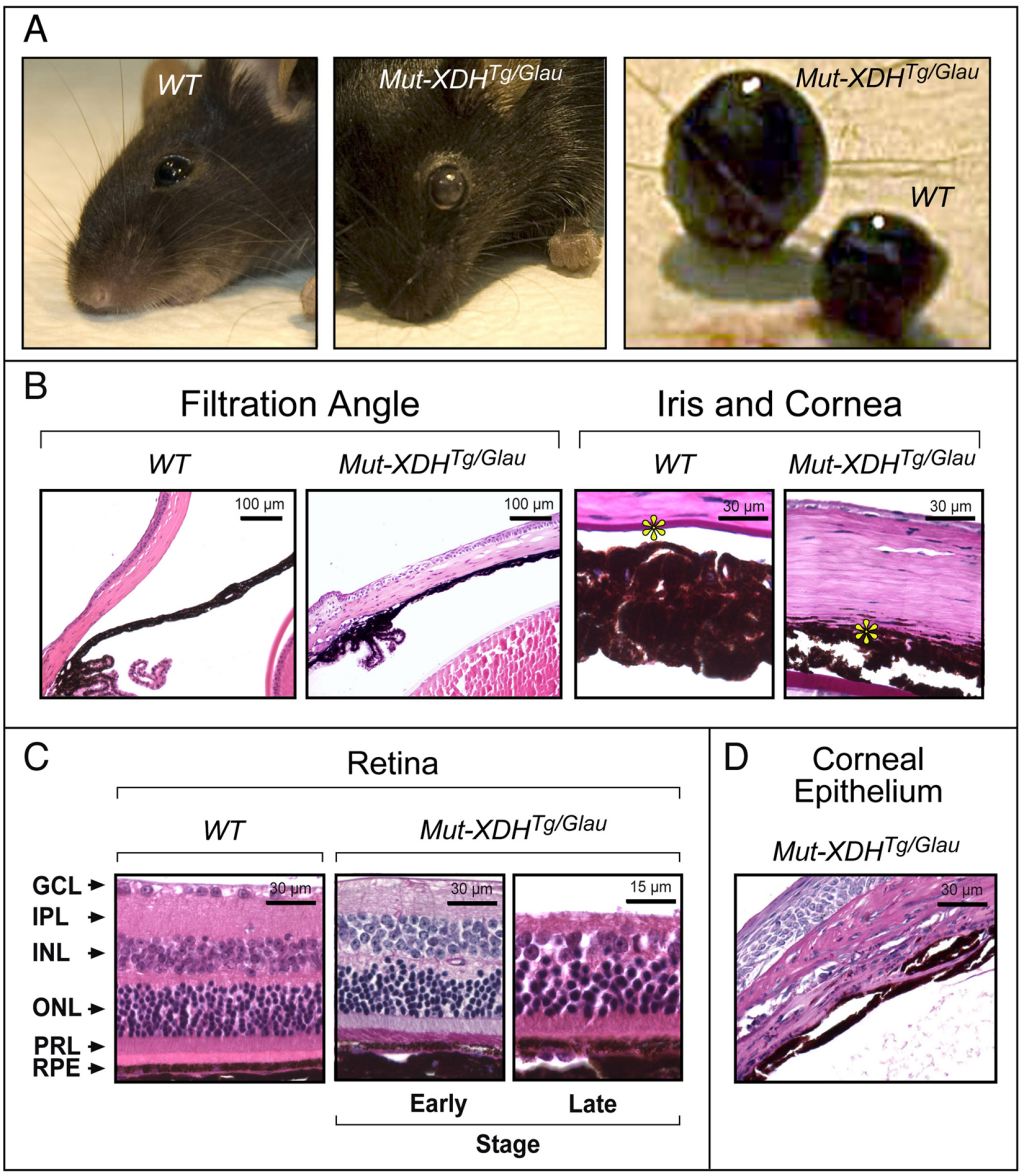
The glaucomatous phenotype of transgenic animals. (**A**) The two leftmost panels illustrate the gross phenotype of a representative transgenic mouse with glaucoma (*Mut*-*XDH* ^*Tg*/*Glau*^) as compared to a parental wild-type (*WT*) control. The rightmost panel shows buphtalmos of the glaucomatous eye. (**B**) and (**C**) The panels show the histology of the indicated sections of the eyes obtained from representative *WT* and *Mut*-*XDH* ^*Tg*/*Glau*^ animals. (**B**) *Anterior Chamber and Filtration angle* (Filtration Angle, Hematoxilin-Eosin staining): *WT*, the panel shows a normal shaped filtration angle; *Mut*-*XDH* ^*Tg*/*Glau*^, the panel shows a closed and collapsed filtration angle. *Anterior Segment Architecture* (Iris and Cornea, PAS staining): *WT*, the picture shows normal iris and Descemet membrane. The iris is not adherent to the cornea and the Descemet membrane is easily recognizable (yellow star); *Mut*-*XDH* ^*Tg*/*Glau*^, the panel indicates that the iris and deep corneal stroma merge diffusely. (**C**) *Retina* (Hematoxilin-Eosin staining): *WT*, the panel illustrates the characteristic layers of a normal retina; *Mut*-*XDH* ^*Tg*/*Glau*^, the two panels show the structure of the retina in two glaucomatous mice representing different severity of the atrophic process. Early stage = the ganglion cells are no longer recognizable; Late stage = a severe atrophy of inner retinal layers is evident, while the photoreceptors are still recognizable. RPE = retinal pigment epithelium; ONL = outer nuclear layer; PRL = photoreceptor layer; IPL = inner plexiform layer; GCL = Ganglion cell layer. (**D**) The panel illustrates some of the corneal alterations observed in the glaucomatous eyes of a representative *Mut*-*XDH* ^*Tg*/*Glau*^ mouse. Histochemical evaluation shows severe thickening and keratosis of the corneal epithelium, fibrosis, neovascularization and inflammatory infiltration of the stroma (exposure keratitis) after PAS staining. All the analyses illustrated in the figure were performed on female animals of 12 months of age.

**Figure 3:**
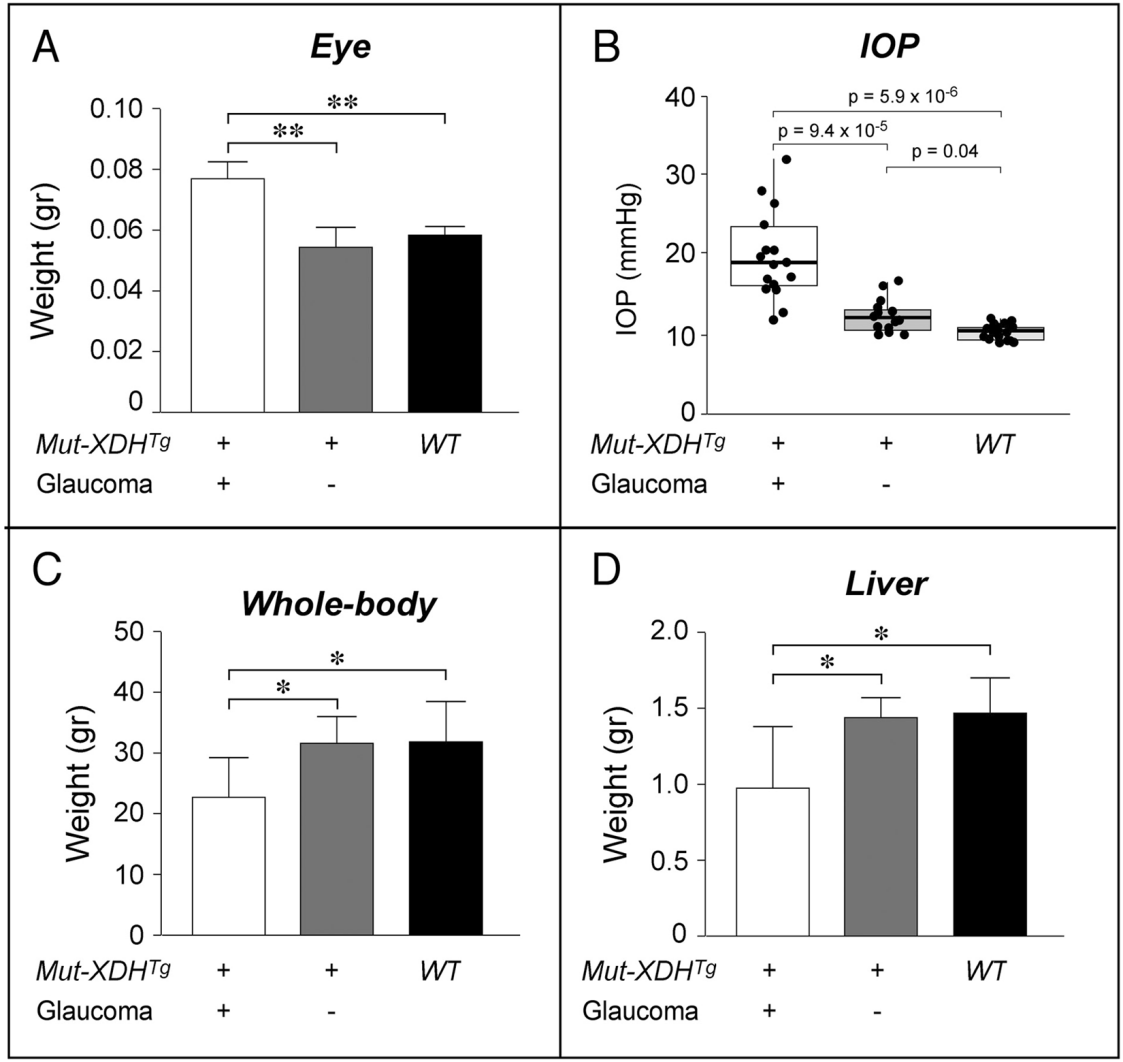
Eye, whole-body and liver weight of WT and transgenic mice. (**A**) The panel illustrates the eye, whole-body and liver weights of *WT*, non-glaucomatous and glaucomatous adult *Mut*-*XDH* transgenic animals (*Mut*-*XDH* ^*Tg*^). (**B**) The values of intra-ocular pressure (IOP) measured in each eye of the indicated type of animals are illustrated. A mean of 3 measurements for each eye was performed and each measurement was repeated 10 times to obtain the overall mean. Total no. of animals used: *WT* = 9; *Mut*-*XDH* ^*Tg*/*Norm*^ = 7; male *Mut*-*XDH* ^*Tg*/*Glau*^ = 8. The thick horizontal lines of each box plot represents the calculated median value, while the vertical lines indicate the distribution of the values. The p-value of the indicated comparisons following Student’s t-test analysis is shown. (**C**) and (**D**) The whole-body and liver weights of *WT*, non-glaucomatous and glaucomatous adult *Mut*-*XDH* transgenic animals (*Mut*-*XDH* ^*Tg*^) are shown in the two panels. All the analyses were performed in animals stabilized on the C57BL/6J genetic background (approximately 1 year of age). The number of animals analyzed is the following: *WT* = 7; male *Mut*-*XDH* ^*Tg*/*Glau*^ = 7; *Mut*-*XDH* ^*Tg*/*Norm*^ = 5.

During the course of functional studies on the mouse molybdo-flavoenzyme, xanthine dehydrogenase (XDH)^12–16, 26^, we generated 6 independent transgenic lines with stable insertion of an expression plasmid (Fig. 1A) containing the cDNA coding for a R883/M mutant of the XDH protein (*Mut*-*XDH*), which is characterized by acquisition of a different substrate specificity relative to the parental enzyme^21^. Indeed, *Mut*-*XDH* is endowed with reduced hypoxanthine oxidase activity and an increased ability to oxidize phthalazine (Fig. 1B). The original idea was to inhibit endogenous XDH, *via* a dominant negative effect, as the active form of the enzyme is a homodimeric protein^19, 27^. Following successive rounds of interbreeding, we observed that approximately 20% of the animals deriving from one of the transgenic lines was characterized by diffuse corneal opacity and increased volume of the eye globe (buphthalmos), which became evident after weaning (Fig. 2A). Regardless of the sex, adult pathologic mice showed buphthalmos and corneal edema (grossly suggestive of glaucoma) and, in some cases, this was associated with signs of keratitis. The glaucomatous phenotype was supported by histological studies performed in the eyes of pathologic (*Mut*-*XDH* ^*Tg*/*Glau*^) and wild-type (*WT*) animals (Fig. 2B and C). No significant pathologic traits were detectable in the eye of *WT* (Fig. 2B and C) and non-glaucomatous transgenic (*Mut*-*XDH* ^*Tg*/*Norm*^) animals. Histologically, the eyes of *Mut*-*XDH* ^*Tg*/*Glau*^ mice, were characterized by complete closure of the filtration angle, whose architecture was no longer recognizable (Fig. 2B). The iris was adherent to the cornea, which lacked the corneal endothelium and Descemet membrane. This was accompanied by diffuse merging of the iris stroma with the deepest corneal stroma (Fig. 2B). Corneal lesions consistent with exposure keratitis (epithelium ulceration and keratosis as well as neutrophilic inflammatory infiltration in the outer stroma) were also present in some animals (Fig. 2D). Signs of neuroretinal atrophy were always evident (Fig. 2C, middle) and they progressed from the inner layers to full thickness retinal atrophy (panretinal atrophy; Fig. 2C, right). A different distribution of the severity of degeneration between different segments of the retina was frequently observed. Overall, the histological findings were consistent with defective development of the anterior segment (anterior segment dysgenesis) and filtration angle closure leading to glaucoma. As expected, relative to both *WT* or *Mut*-*XDH* ^*Tg*/*Norm*^ animals, the eyes of adult and pathologic *Mut*-*XDH* ^*Tg*/*Glau*^ mice were characterized by an increase in weight (Fig. 3A). Given this general picture, we measured the intra-ocular pressure (IOP) of *WT*, *Mut*-*XDH* ^*Tg*/*Norm*^ and *Mut*-*XDH* ^*Tg*/*Glau*^, as an increase in this parameter is considered to be a major feature of glaucoma in various animal species (Fig. 3B). The results obtained in adult mice indicated that the average IOP values of *Mut*-*XDH* ^*Tg*/*Glau*^ mice were significantly higher than the ones observed in the *WT* and *Mut*-*XDH* ^*Tg*/*Norm*^ counterparts. Indeed the average IOP of *Mut*-*XDH* ^*Tg*/*Glau*^ mice was around 20 mmHg. *Mut*-*XDH* ^*Tg*/*Norm*^ mice showed a significant though modest increase in IOP relative to *WT* animals. The elevated IOP values observed in pathologic animals were entirely consistent with glaucoma. In these animals, increased size of the eyes and elevated IOP values were accompanied by a reduction in the size and weight of the body (Fig. 3C) and peripheral tissues, such as the liver (Fig. 3D), which was indicative of a mild developmental deficit. Noticeably, long-term observation of our animal colonies indicated that *Mut*-*XDH* ^*Tg*/*Glau*^ mice did not show major alterations in motor activity or feeding behavior, which may be at the basis of the reduction in size and weight. As for the feeding behavior, the daily food-intake of adult *WT*, *Mut*-*XDH* ^*Tg*/*Norm*^ and *Mut*-*XDH* ^*Tg*/*Glau*^ mice, calculated over a period of one month, did not show any statistically significant difference (data not shown).

### Expression of the Mut-XDH transgene is unlikely to explain the glaucomatous phenotype

**Figure 4:**
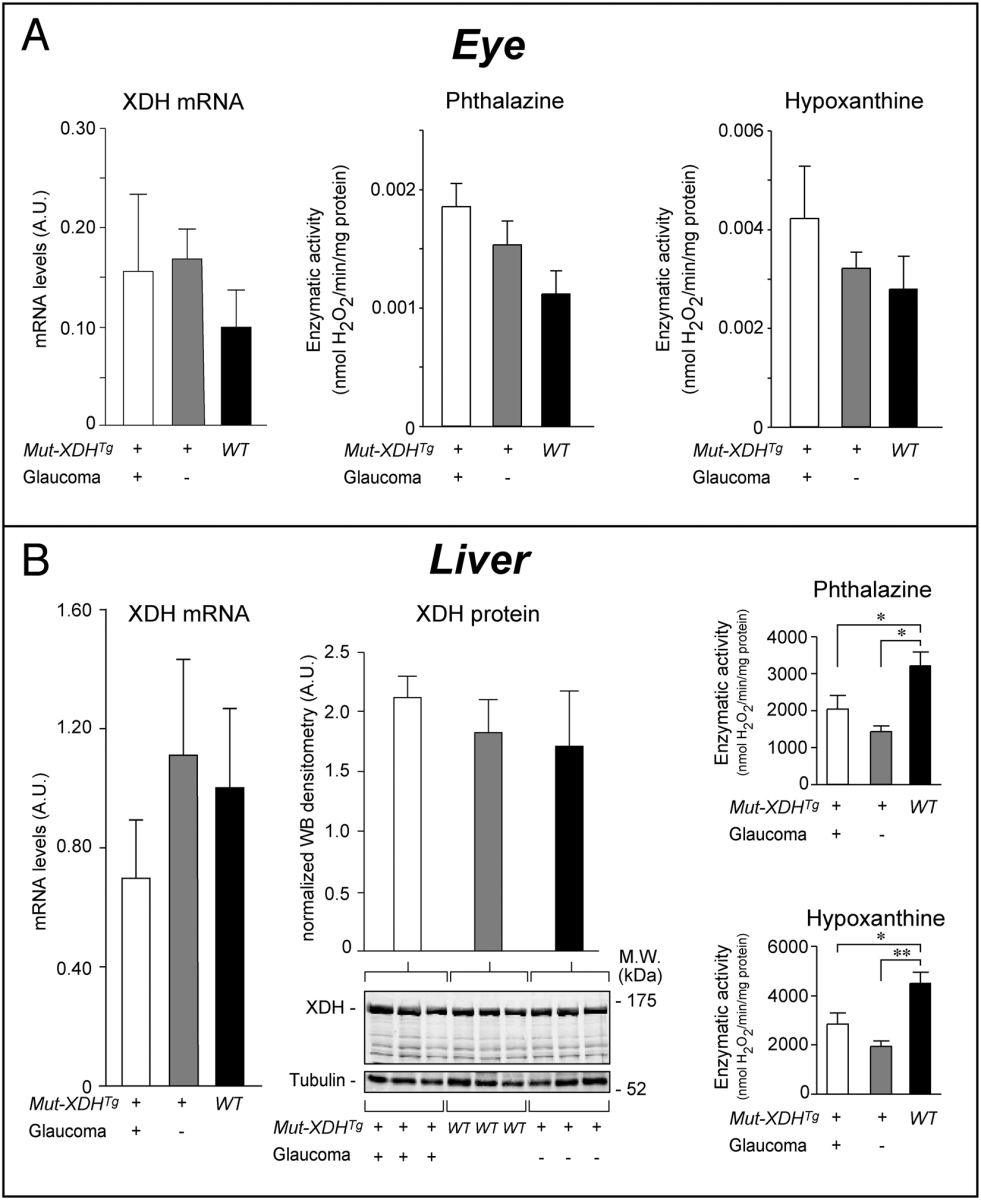
Levels of the XDH mRNA, protein and enzymatic activity in the eye and liver of WT and transgenic mice. Total RNA and protein extracts obtained from the eye (**A**) and liver (**B**) of *WT*, non-glaucomatous and glaucomatous *Mut*-*XDH* ^*Tg*^ animals were used to measure the levels of the XDH mRNA and enzymatic activity. The XDH protein levels were determined by Western blot analysis in liver. As for the XDH mRNA (leftmost diagram), each value represents the mean ± SD of 4 female animals. As for the XDH protein (middle panels) each lane of the Western blot represents a single liver. The same amount of protein was loaded in each lane, as indicated by the Western blot signal of tubulin. The graph is the mean ± SD of the densitometric analysis of the XDH band following normalization for the tubulin signal. As for XDH enzymatic activity (2 rightmost diagrams), each value is the mean ± SD of 3 female animals. Enzymatic activity was measured using either phthalazine or hypoxanthine as substrates. *Significantly different (p < 0.05) after Student’s t-test. **Significantly different (p < 0.01) after Student’s t-test. The full-length blots/gels are presented in Supplementary Figure S1.

To establish whether expression of *Mut*-*XDH* is involved in the generation of glaucoma, we measured the levels of XDH mRNA, protein and phthalazine/hypoxanthine oxidizing activity in the eye and liver of female *WT*, *Mut*-*XDH* ^*Tg*/*Glau*^ and *Mut*-*XDH* ^*Tg*/*Norm*^ mice. In the eye, the amounts of XDH mRNA were just measurable and similar in *WT*, *Mut*-*XDH* ^*Tg*/*Glau*^ and *Mut*-*XDH* ^*Tg*/*Norm*^ animals (Fig. 4A). The same was true in the case of XDH enzymatic activity, which was determined with phtalazine and hypoxanthine as substrates. The levels of both phthalazine and hypoxanthine oxidizing activity were just above the detection limit and showed no statistically significant difference in the three types of animals. Regardless of the presence/absence of glaucoma, a specific XDH protein band in either *WT* or transgenic animals could not be detected upon Western blot analysis. Similar results were observed in the eyes of male animals. As the promoter (β-actin) controlling the transgene is ubiquitous, we measured XDH mRNA, protein and activity in female mice liver (Fig. 4B). The levels of XDH mRNA did not differ in *WT*, *Mut*-*XDH* ^*Tg*/*Norm*^ and *Mut*-*XDH* ^*Tg*/*Glau*^ animals. In the three types of mice, similar amounts of the XDH protein were evident. Relative to the *WT* counterpart, the levels of liver phthalazine and hypoxanthine oxidizing activity were similarly reduced in both *Mut*-*XDH* ^*Tg*/*Norm*^ and *Mut*-*XDH* ^*Tg*/*Glau*^ mice. The functional significance of this last observation is currently unknown, although the finding is consistent with the original hypothesis of a dominant negative effect exerted by the *Mut*-*XDH* protein on the native enzyme. Nevertheless, this minor effect and the results obtained on XDH in the eye are unlikely to explain the insurgence of glaucoma. This along with the appearance of glaucoma in only one of the 6 transgenic lines generated, supported the hypothesis that the pathologic phenotype was not due to expression of the *Mut*-*XDH* transgene, but rather to its insertion into a critical region of the genome.

### Associations between the glaucomatous phenotype and the Mut-XDH transgene

To evaluate whether the glaucomatous phenotype is related to segregation of the transgene, we measured the presence/absence of the *Mut*-*XDH* transgene in the colony of *B6D2F1* mice, with a PCR assay which did not distinguish between transgene heterozygosity and homozygosity (Table 1). 73% of the animals were *Mut*-*XDH* positive, which is consistent with a mendelian transmission of the transgenic allele, as these mice represent the sum of the heterozygous and homozygous animals. As for the pathologic phenotype, 19% of the mice were glaucomatous and all of them were *Mut*-*XDH* positive. This was significantly different from the expected proportion of animals homozygous for the transgene, which is consistent with an incomplete penetrance of the trait under the assumption that the glaucomatous phenotype is associated to transgene homozygosity. Sex exerted only a marginal influence on the frequency of glaucoma.

**Table 1:** Transmission of the Mut-XDH transgene and the glaucomatous phenotype in the colony of B6D2F1 mice.

B6D2F1 mice	Mut-XdhTg−Normal (A)	Mut-XdhTg^+^ Normal (B)	Chi-square p-valHypothesis (A): 25% (B + C): 75%	Chi-square p-valHypothesis (A): 25% (B): 50% (C): 25%
MALE	67 (30%)	110 (50%)	0.07	0.09
FEMALE	51 (24%)	124 (59%)	0.75	0.02
MALE + FEMALE	118 (27%)	234 (54%)	0.28	0.01

Transgenic mice deriving from the founder animal and stabilized on the *B6D2F1* background were crossed for a number of generations and the number of animals positive (*Mut*-*Xdh* ^+^) or negative (*Mut*-*Xdh* ^−^) for the transgene by PCR and characterized by the presence (*Glaucoma*) or the absence (*Normal*) of the glaucomatous phenotype were identified. As the PCR assay does not distinguish between heterozygosity and homozygosity for the transgene, the values reported in column 3 represent the sum of heterozygous and homozygous animals. Column 6 illustrates the p-values (*p*-*val*) of the comparisons between the observed frequency of *Mut*-*XDH* positivity and the values expected on the basis of a mendelian transmission of the transgene. Column 5 illustrates the p-values (*p*-*val*) of the comparisons between the observed frequency of *Mut*-*Xdh* ^−^, *Mut*-*Xdh* ^+^ *Normal* and *Mut*-*Xdh* ^+^ *Glaucoma* under the hypothesis that all homozygous transgenic animals show the glaucomatous phenotype.

The genetic background may modulate the insurgence of glaucoma, as *Mut*-*XDH* transgenic mice were originally generated in the *B6D2F1* hybrid strain (*C57BL*/*6J* X *DBA*/*2*) and *DBA*/*2* mice are known to develop late-onset glaucoma^28^. Thus, we stabilized our transgenic animals on the *C57BL*/*6J* background by over 10 successive rounds of breeding. Overall, 26% of the *C57BL*/*6J* animals were negative, while the remainder were positive for the transgene, consistent with a mendelian segregation (Table 2). The frequency of glaucoma in *C57BL*/*6J* and *B6D2F1* mice was almost identical (18% vs. 19%). All glaucomatous *C57BL*/*6J* mice were positive for the transgene and the frequency of glaucoma was similar in the female and male populations.

**Table 2:** Transmission of the Mut-XDH transgene and the glaucomatous phenotype in the colony of C57Bl/6J mice.

C57Bl/6J mice	Mut-XdhTg^+^ Total (B + C)	Mut-XdhTg^+^ Normal (B)	Mut-XdhTg^+^ Glaucoma (C)	Chi-square p-val Hypothesis (A): 25%, (B + C): 75%	Chi-square p-val Hypothesis (A): 25% (B): 50% (C): 25%
MALE	101 (72%)	76 (54%)	25 (18%)	0.43	0.15
FEMALE	92 (77%)	69 (57%)	23 (19%)	0.67	0.21
MALE + FEMALE	193 (74%)	145 (56%)	48 (18%)	0.77	0.04

Transgenic mice deriving from the founder animal and stabilized on the *C57Bl*/*6J* background were inter-crossed for a number of generations and the number of animals positive (*Mut*-*Xdh* ^+^) or negative (*Mut*-*Xdh* ^−^) for the transgene by PCR and characterized by the presence (*Glaucoma*) or absence (*Normal*) of the glaucomatous phenotype were identified. As the PCR assay does not distinguish between heterozygosity and homozygosity for the transgene, the values reported in column 3 represent the sum of heterozygous and homozygous animals. Column 6 illustrates the p-values (*p*-*val*) of the comparisons between the observed frequency of *Mut*-*XDH* positivity and the values expected on the basis of a mendelian transmission of the transgene. Column 6 illustrates the p-values (*p*-*val*) of the comparisons between the observed frequency of *Mut*-*Xdh* ^−^, *Mut*-*Xdh* ^+^ *Normal* and *Mut*-*Xdh* ^+^ *Glaucoma* under the hypothesis that all homozygous transgenic animals show the glaucomatous phenotype.

### Genomic sequencing defines a complex insertion site of the Mut-XDH transgene on Chromosome 1

**Figure 5:**
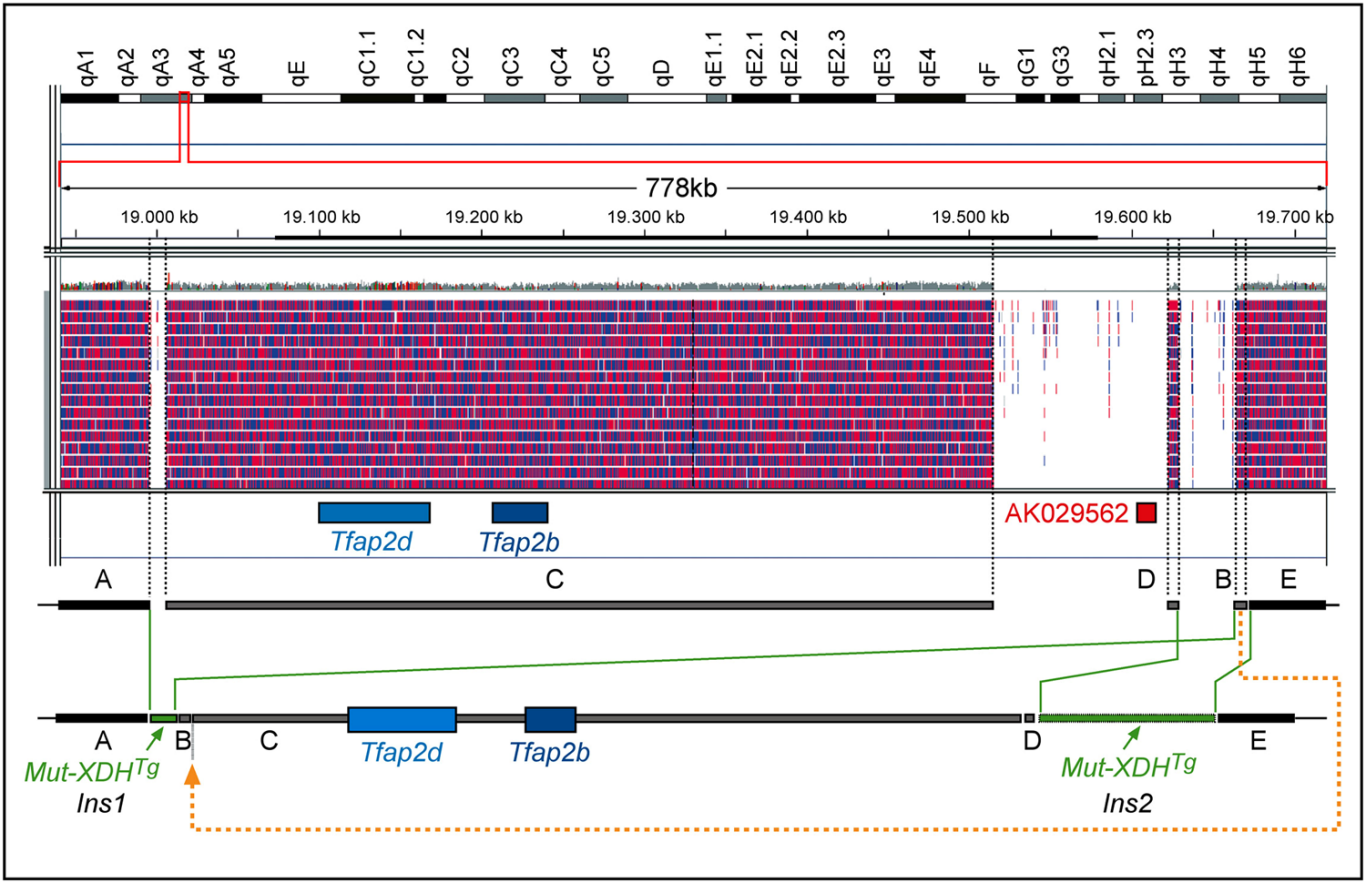
The genome insertion site of the Mut-XDH transgene. A schematic representation of the insertion site of the *Mut*-*XDH* transgene (*Mut*-*XDH* ^*Tg*^) into the indicated region of chromosome 1qA3, as determined upon genomic sequencing of a glaucomatous *Mut*-*XDH* ^*Tg*/*Glau*^ mouse is shown. The sequenced fragments and the corresponding alignments are indicated by the red and blue vertical lines. The inserted transgene fragments are indicated by the green boxes. The position of the two genes *Tfap2b* and *Tfap2d* coding for the AP-2β and AP-2δ proteins, respectively, as well as the non-coding *AK029562* gene are indicated by the boxes drawn in different colors. *Ins1* and *Ins2* = insertion sites 1 and 2. The position of the genomic regions (**A**–**E**) in the *WT* and the glaucomatous *Mut*-*Xdh* ^+/+^ animal are shown by the upper and lower grey or black boxes, respectively.

**Figure 6:**
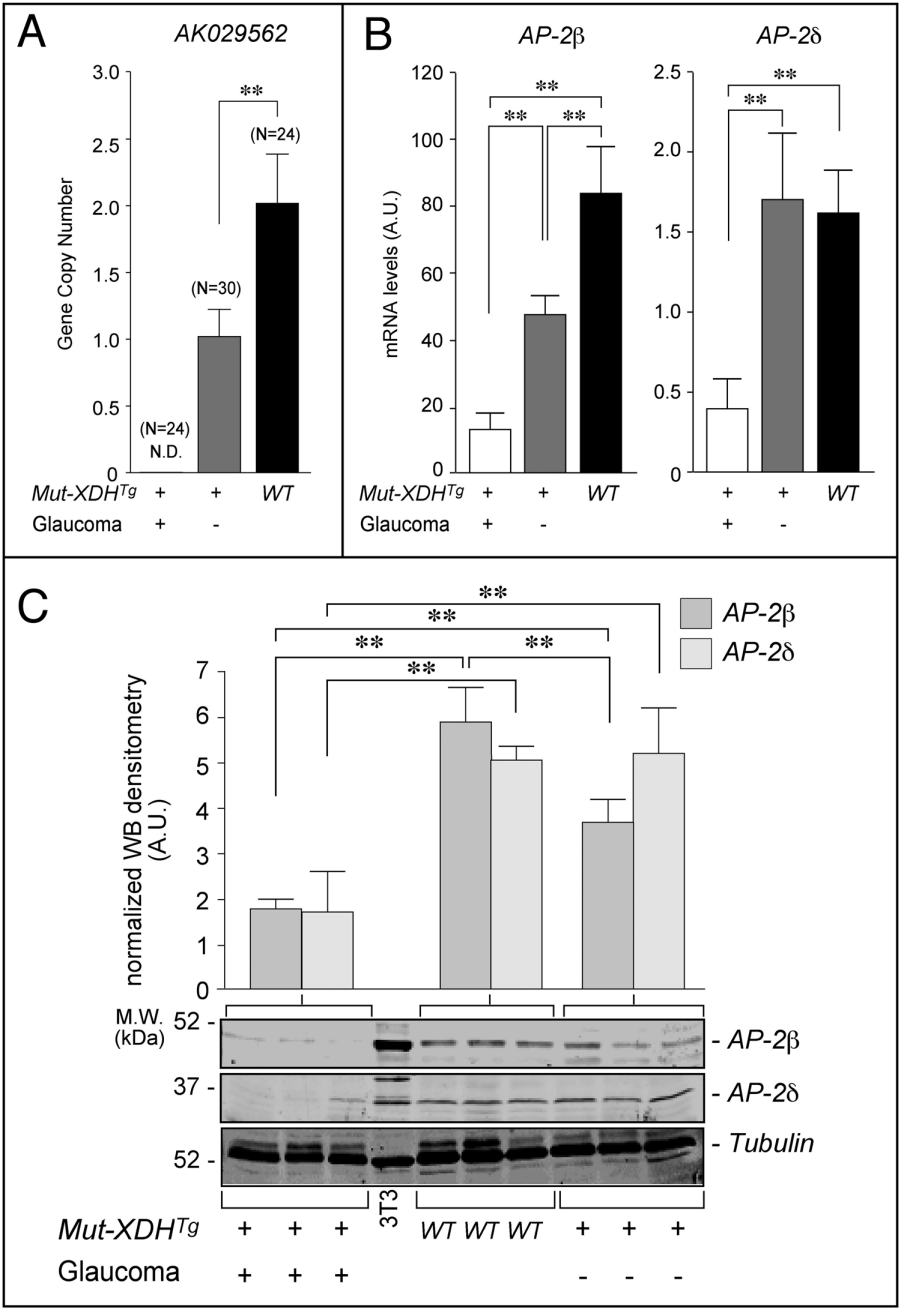
Copy number of the *AK029562* gene as well as AP-2β/AP-2δ mRNA and protein levels in the eyes of WT and transgenic animals. (**A**) The panel illustrates the copy number of the *AK029562* gene in *WT*, non-glaucomatous *Mut*-*XDH* ^*Tg*^ and glaucomatous *Mut*-*XDH* ^*Tg*^ mice. **Significantly different (p < 0.01) after Student’s t-test. N = the number of animals analyzed. Each value is expressed as the Mean ± SD. (**B**) The expression levels of the AP-2β and AP-2δ mRNAs in *WT*, non-glaucomatous *Mut*-*XDH* ^*Tg*^ as well as glaucomatous *Mut*-*XDH* ^*Tg*^ animals are indicated. Each value is the mean ± SD of 7 distinct animals. **Significantly different (p < 0.01) after Student’s t-test. (**C**) The amounts of AP-2β and AP-2δ proteins in the eyes of *WT*, non-glaucomatous *Mut*-*XDH* ^*Tg*^ and glaucomatous *Mut*-*XDH* ^*Tg*^ animals were measured by Western blot analysis. Each lane represents a single animal. The upper bar graphs illustrate the results obtained after densitometric analysis of the AP-2β and AP-2δ bands and normalization for the tubulin band. **Significantly different (p < 0.01) after Student’s t-test. The full-length blots/gels are presented in Supplementary Figure S2.

As our data indicated that *Mut*-*XDH* expression was unlikely to be responsible for the glaucomatous phenotype, we defined the integration site of the transgene. We sequenced the whole genome of a representative *Mut*-*XDH* ^*Tg*/*Glau*^ animal and demonstrated a unique insertion of the transgenic construct into Chr1qA3 (Fig. 5). The insertion consists of two separate sub-sites containing different parts of the transgenic construct. Indeed, multiple copies of the vast majority of the *Mut*-*XDH* construct are located approximately 250 kilobases downstream of the two coding genes (*Tfap2b* and *Tfap2d*) present in this genomic region (*Ins2*). The other sub-site (*Ins1*) maps 100 Kb upstream of the *Tfap2d* locus and contains a small fragment of *Mut*-*XDH* (517 bases). The insertion sub-sites are associated with 3 genomic deletions. The largest deletion (100 kb) involves *AK029562*, which is an ill characterized gene. It is noticeable that the gene is not expressed in the eye, as indicated by measurement of the corresponding mRNA by PCR analysis in *WT* animals. Nevertheless, we exploited the predicted *AK029562* deletion to support the hypothesis that the glaucomatous phenotype segregates with transgene homozygosity. Thus, we defined the copy-number of the *AK029562* gene in the colony of *C57BL*/*6J* mice (Fig. 6A). All the *WT* animals presented with two copies of the *AK029562* gene, while a single copy was present in *Mut*-*XDH* ^*Tg*/*Norm*^ mice. The *AK029562* gene was undetectable in glaucomatous *Mut*-*XDH* ^*Tg*/*Glau*^ mice. The results obtained demonstrate an association between transgene homozygosity and glaucoma.

### Mut-XDH transgene insertion is associated with a decrease in the eye levels of AP-2β mRNA and protein

**Figure 7:**
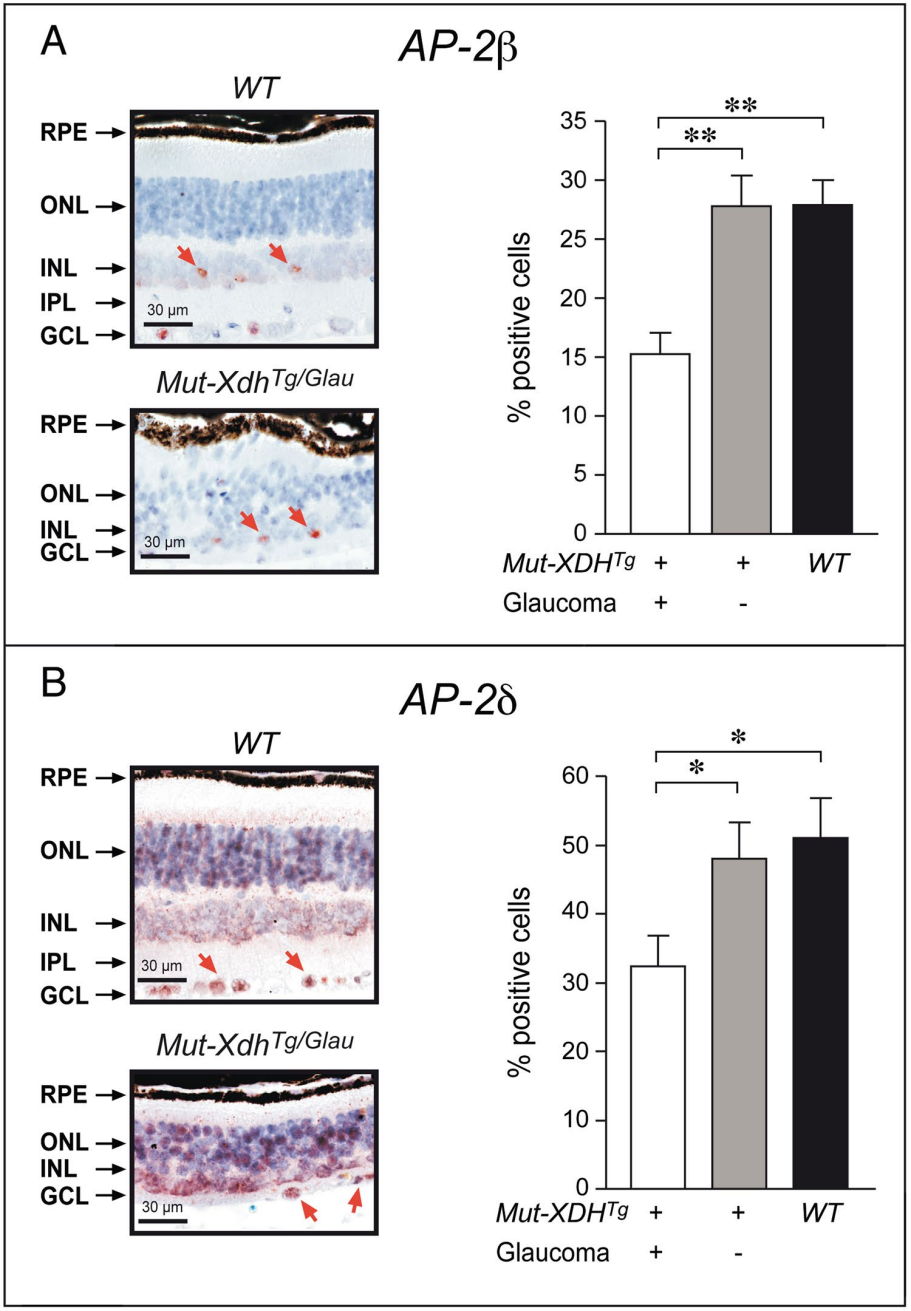
Immunohistochemical analysis of AP-2β and AP-2δ in the retina of *WT* and transgenic animals. The retinas of wild-type (*WT*) and glaucomatous *Mut*-*XDH* ^*Tg*/*Glau*^ mice were subjected to immunohistochemical analysis using anti- AP-2β (**A**) and anti-AP-2δ (**B**) antibodies. The number of AP-2β- and AP-2δ-positive cells was evaluated under the microscope in 5 HPF fields/eye and 4 distinct animals/experimental group. Each value is the Mean +/- SE of at least 15 experimental point. Left: Representative retinal fields from one *WT* and one *Mut*-*XDH* ^*Tg*/*Glau*^ mouse. Right: Quantitative measurement of the number of AP-2β- and AP-2δ-positive cells in the indicated groups of mice. RPE = retinal pigment epithelium; ONL = outer nuclear layer; INL = inner nuclear layer; IPL = inner plexiform layer; GCL = Ganglion cell layer.

*Tfap2b* and *Tfap2d*, which express the AP-2β and AP-2δ proteins, respectively, are the only coding genes proximal to the *Mut*-*XDH* insertion site. These genes encode two transcription factors belonging to the family of activating protein 2 (AP-2) which consists of 5 members (AP-2α, AP-2β, AP-2γ, AP-2δ and AP-2ε)^29^. AP-2 transcription factors are known to play an important role in eye development^30–33^. Recently, conditional deletion of the *Tfap2b* gene in the neural crest was shown to cause defective development of the eye anterior segment and early-onset glaucoma^10^. In these conditional knock-out and our *Mut*-*XDH* ^*Tg*/*Glau*^ mice, the morphological/histological features of the glaucomatous pathology are similar. Thus, we evaluated whether *Mut*-*XDH* insertion caused changes in the eye levels of AP-2β and AP-2δ transcripts/proteins. Relative to *WT* animals, the eyes of *Mut*-*XDH* ^*Tg*/*Norm*^ mice expressed significantly smaller amounts of the AP-2β mRNA, while no difference was evident in the case of AP-2δ. AP-2β and AP-2δ mRNAs were substantially and similarly down-regulated in the eyes of *Mut*-*XDH* ^*Tg*/*Glau*^ mice (Fig. 6B). These data were confirmed at the protein level by Western blot analysis (Fig. 6C). As AP-2β and AP-2δ are predominantly expressed in different cellular components of the developing and adult retina^33, 34^, we determined the amounts of the two proteins in this tissue by quantitative immuno-histochemistry. The results obtained demonstrated that the number of cells positive for AP-2β and AP-2δ was the same in the retina of *WT* and *Mut*-*XDH* ^*Tg*/*Norm*^ mice (Fig. 7A and B). Relative to both *WT* and *Mut*-*XDH* ^*Tg*/*Norm*^ mice, a substantial decrease in the number of AP-2β and AP-2δ positive cells was evident in *Mut*-*XDH* ^*Tg*/*Glau*^ animals. The collected evidence suggests that down-regulation of AP-2δ mRNA and protein, which is obvious only in glaucomatous *Mut*-*XDH* ^*Tg*/*Glau*^ mice, is secondary to the retinal atrophy induced by transgene homozygosity. In contrast, the decrease in AP-2β mRNA and protein which is already observed in non-pathologic *Mut*-*XDH* ^*Tg*/*Norm*^ mice, may precede and cause glaucoma, which would subsequently evolve in retinal atrophy. In *Mut*-*XDH* ^*Tg*/*Glau*^ animals, we propose that this event reduces, but does not abolish the synthesis of the AP-2β protein, as complete ablation of the *Tfap2b* gene would result in perinatal death^35^.

## Discussion

Glaucoma is a heterogeneous group of progressive disorders and a major cause of blindness, characterized by retinal and optic nerve degeneration. Increased IOP is considered to be an important risk factor for glaucoma. The mechanisms underlying the progression of glaucoma are still largely unknown and this represents a major obstacle for the development of efficacious therapeutic strategies. A possible solution to the problem is represented by the development of new and manageable animal models of the disease. In this study, we describe the generation of a transgenic mouse characterized by glaucoma which eventually develops into sight loss as indicated by our histological analyses. Indeed, our data provide evidence of progressive retinal atrophy in adult glaucomatous transgenic mice. The glaucomatous phenotype is associated to homozygous segregation of the transgene, as indicated by the results obtained after long term breeding of our transgenic colony. The penetrance of glaucoma in the transgene homozygotes is approximately 75% and the pathologic trait is very stable, being observed with the same frequency across more than 10 generations. The frequency of the glaucomatous phenotype is not significantly influenced by the genetic background. In fact, a similar proportion of glaucomatous animals is observed in the two mouse strains on which the transgene has been stabilized. In addition, sex is another factor that does not play a role in the incidence of glaucoma in our transgenic animals. Taken together, these observations support the idea that the glaucomatous phenotype is a direct consequence of transgene expression or transgene insertion into the mouse genome. Our data are in line with the second hypothesis, as the *Mut*-*XDH* transgene, encoding a specific amino acid mutant of the XDH molybdo-flavoprotein^16^ is not expressed in the eye at detectable levels. In addition, the minor reduction in the amounts of XDH enzymatic activity observed in the liver of heterozygous and homozygous transgenic animals is difficult to interpret as for its possible relevance in the etio-pathogenesis of the glaucomatous trait. A strong argument against the hypothesis that expression of the *Mut*-*XDH* transgene is relevant comes from our genome sequencing experiments, which favor the idea that the transgene insertion site is a critical determinant of the glaucomatous phenotype. In fact, the single, though complex, insertion site maps in close proximity to the *Tfap2b* and *Tfap2d* coding genes. *Tfap2b* and *Tfap2d* code for the AP-2β and AP-2δ transcription factors, respectively, which are known to be involved in the development and function of the neural crest in vertebrates^11^. In addition, AP-2β can influence various aspects of eye development, given its activity in the surface ectoderm and neural retina^33^. More importantly, while constitutive knock-out of the same gene is lethal^35^, conditional knock-out of the *Tfap2b* in the cranial neural crest results in early-onset glaucoma^10^. Interestingly, the macroscopic and microscopic characteristics of the glaucomatous phenotype described in the *Tfap2b* conditional knock-out and our transgenic animal are very similar. The observation strongly supports the idea that the significant down-regulation of *Tfap2b* gene expression observed in our transgenic animals is causally related to the appearance of glaucoma. With respect to this point, it would be of interest to compare the gene expression profiles of the retina and other eye components in developing *WT*, *Mut*-*XDH* ^*Tg*/*Glau*^ and *Mut*-*XDH* ^*Tg*/*Norm*^ mice with the aim of identifying the gene-networks affected by TFAP-2β. In prospective, a comparison of these data with the results of similar studies performed in *Tfap2b* conditional knock-out animals may provide further information.

A substantial decrease of AP-2β mRNA and protein is already evident in heterozygous and non-glaucomatous transgenic mice. This, along with the observation that the structure of the gene is unaffected, strengthens the idea that the transgene insertion site falls within a genomic region which regulates the *Tfap2b* transcription. In contrast, the decrease in the eye levels of AP-2δ is likely to be secondary to the retinal atrophy induced by the glaucoma. These considerations support the contention that down-regulation of AP-2β, rather than AP-2δ, is at the basis of the glaucomatous phenotype observed in our transgenic mice. At present the molecular mechanisms underlying the reduction of *Tfap2b* by the insertion of the *Mut*-*XDH* transgene are unknown. Nevertheless, we can rule out a direct effect of the transgene product on AP-2β mRNA and protein expression. In fact, transient *Mut*-*XDH* over-expression in NIH3T3 cells, which synthesize detectable levels AP-2β and AP-2δ, has no effect on the expression of the corresponding mRNAs or proteins (MT, unpublished results). We propose that the *Mut*-*XDH* insertion site alters the structure and activity of an enhancer region upstream or downstream the *Tfap2b* locus, which reduces the transcription of this gene. With respect to this, it is important to notice that the *Mut*-*XDH* transgene interrupts the *AK029562* non-coding gene, which is potentially transcribed into a long inter-dispersed RNA. However, inactivation of the AK029562 RNA is unlikely to play a role in the reduced transcription of the *Tfap2b* gene. In fact, the AK029562 mRNA is not detectable in the eyes of either *WT* or glaucomatous transgenic animals.

Overall the results of the study represent independent evidence that *Tfap2b* is involved in the etio-pathogenesis and progression of glaucoma^10^. With respect to this, it is worth noticing that our data indicate that full suppression of *Tfap2b* gene expression is not necessary to cause a deficit in the development of the anterior segment of the eye which results in angle-closure glaucoma. Indeed, they indicate that a decrease in the levels of AP-2β below a certain threshold, as observed in the case of our homozygous transgenic animals, is sufficient to cause glaucoma. Thus, it is possible to speculate that a quantitative reduction in the amounts of AP-2β favors the progression of glaucoma, which may have implications from a therapeutic perspective. In fact, it suggests that AP-2β may represent a novel pharmacological target of intervention and strategies aimed at restoring a proper expression of the *Tfap2b* is of therapeutic interest. In terms of its potential use in pre-clinical studies aimed at developing new therapeutic strategies, our transgenic mouse has some advantages over the few spontaneous or genetic models of glaucoma currently available, such as the consistency and frequency of the glaucomatous trait as well as the relatively short time necessary to induce retinal degeneration. Indeed, animal models, like the DBA/2 J inbred strain^36^ and the myocilin^37^ or the α1 subunit of collagen type I transgenic mice^38^, show retinal degeneration after 6–12 months. In addition these models show a high variability in the incidence and characteristics of the glaucomatous trait and studies based on them require large cohorts of animals.

*Mut*-*XDH* ^*Tg*/*Glau*^ mice are also characterized by similarities and differences relative to the model of glaucoma generated by Martino *et al*. and based on conditional silencing of the *Tfap2b* gene in the cranial neural crest^10^. The two types of genetically engineered animals have distinct advantages and disadvantages in terms of their potential as experimental models to conduct basic studies aimed at defining the regulation and physiological function of AP-2β, with particular reference to the role played by the protein in the genesis of certain types of glaucoma. Indeed, the conditional knock-out animals are characterized by a higher frequency and penetrance of glaucoma than *Mut*-*XDH* ^*Tg*/*Glau*^ mice. Thus, an intrinsic and practical advantage of the conditional knock-out mouse is represented by the necessity of a smaller number of animals to obtain statistically significant data. By converse, a distinct benefit of our *Mut*-*XDH* ^*Tg*/*Glau*^ model is given by the fact that glaucoma is a spontaneous event and does not require selective crossing experiments between genetically engineered strains of animals and it does not necessitate any artificial induction system, such as the use of tamoxifen^10^. In addition, it is easy to change the genetic background of *Mut*-*XDH* ^*Tg*/*Glau*^ mice by consecutive mating experiments. This aspect is of extreme importance, given the significant role played by the genetic background in the development of mouse glaucoma^28^. Relative to the conditional knock-out animal, our model is also unique in the context of basic studies aimed at defining the general and tissue-specific mechanisms underlying the regulation of the *Tfap2b* gene. Indeed, the insertion of the transgene in a genomic region involved in the control of *Tfap2b* expression, provides the background for future studies in this direction. In conclusion, our new model of early onset angle-closure glaucoma represents a useful tool for the study of the pathogenesis and the development of innovative therapeutic strategies for this disease.

## Materials and Methods

### Animals

All the procedures involving animals and their care were conducted in conformity with the institutional guidelines in compliance with the national (Legislative Decree n. 26, March 4, 2014; Authorization n.19/2008-A issued March 6, 2008, by the Italian Ministry of Health); Mario Negri Institutional Regulations and Policies providing internal authorization for persons conducting animal experiments (Quality Management System Certificate-UNI EN ISO 9001:2008-Reg.N°6121); EU directives and guidelines (EEC Council Directive 2010/63/EU); the NIH Guide for the care and use of laboratory animals (2011 edition). The statement of Compliance (Assurance) with the Public Health Service (PHS) Policy on Human Care and Use of Laboratory Animals has been recently reviewed (9/9/2014) and will expire on September 30, 2019 (Animal Welfare Assurance #A5023-01. All the animal experiments performed in this study were validated by the Ethical Review Committee for the Animal Care and Use (CESA, Comitato Etico per la Sperimentazione Animale) of the IRCCS-Istituto di Ricerche Farmacologiche “Mario Negri”.

All the mice were housed in individually ventilated cages (IVC) located inside a specific pathogen-free (SPF) animal facility and exposed to environmental enrichment. Animals were subjected to a 12-hours light and 12-hours dark cycle (light phase = 7 am–7 pm) with food (standard chow 2918, Harlan Teklad Global Diet, Madison, WI) and water ad libitum. The animal house facility was maintained in constant environmental conditions at a 22° ± 2 °C temperature and 55 ± 10 RH (relative humidity). Every cage contained five or less mice to ensure comfortable activity space. The IVC were cleaned every week to prevent unnecessary distress or suffering. The physical conditions of the animals were monitored by a member of the veterinary department every 2 days. Cervical dislocation was used as the method of euthanasia for all the animals used in this research.

### Transgenic animals

To generate the transgene, the plasmid construct illustrated in Fig. 1A was first digested with ScaI and subsequently it was partially cleaved with XhoI. The resultant 7 Kb fragment was separated by gel electrophoresis. The isolated fragment was microinjected into the pronuclei of oocytes obtained from female B6D2F1 mice (Charles River Laboratories, Calco, (LC) Italy), which were subsequently implanted into pseudo-pregnant B6D2F1 female mice according to standard methodologies. The animals obtained were screened for the presence of the transgene using Southern blot analysis with a probe specific for the transgene. Positive animals were mated with wild-type *B6D2F1* animals to obtain the F1 generation. Heterozygous transgenic animals were cross-bred to obtain the F2 generation. Transgenic animals were subsequently stabilized on the C57Bl/6J (Envigo, San Pietro al Natisone, Udine, Italy) genetic background by more than 10 rounds of breeding. For each stabilized *B6D2F1* and *C57Bl*/*6J* transgenic line we performed at least 6 matings using 2 female and 1 male positive for the transgene (*Mut*-*Xdh* ^+^). The total number of animals analyzed for the transgene was 433 *B6D2F1* mice and 260 *C57Bl*/*6J* mice. Further details are present in Tables 1 and 2. Genotypic analysis of the transgenic animals colony is routinely performed by PCR analysis using the following amplimers recognizing the transgene: 5′-TTCGGCTTCTGGCGTGTGACCGG-3′ (forward primer, chicken β-actin promoter); 5′-CTCCTCGACAGTAGGCTCAGGCTT-3′ (reverse primer, mouse XDH).

### Measurement of the intra-ocular pressure

Intra-ocular pressure (IOP) measurements were performed on male and female *WT*, *Mut*-*XDH* ^*Tg*^/glaucoma^+^ and *Mut*-*XDH* ^*Tg*^/glaucoma^−^ mice 3 to 7 months of age. Pilot experiments performed in the same conditions demonstrated no significant difference between the IOP of male and female *WT* mice. For this reason, our analyses do not take into account sex and aggregated results are presented (Fig. 3B). A validated commercial rebound tonometer (TonoLab, Vantaa, Finland) with tip positioned at 2–3 mm from the eye^39^ was used for all the measurements. For each animal, the IOP of both eyes was measured. A mean of 3 measurements for each eye was performed and each measurement was repeated 10 times to obtain the overall mean. Measurements were accepted only if the complied to the standards described in the manufacturer’s manual. All measurements on weighed and non-anesthesized animals and were performed between 9.00 and 12.00 p.m. a time of the day characterized by stable IOP according to our experience. Total no. of animals used: male *WT* = 4; female WT = 5; male *Mut*-*XDH* ^*Tg*/ *Glau*^ = 6; female *Mut*-*XDH* ^*Tg/Glau*^ = 1; male *Mut*-*XDH* ^*Tg/Glau*^ = 4; female *Mut*-*XDH* ^*Tg*^/glaucoma^+^ = 5.

### XDH enzymatic activity

XDH enzymatic activity was measured as phthalazine or hypoxanthine oxidizing activity using described protocols^26^. To perform these experiments, the number of animals analyzed was the following: *WT* = 3; male *Mut*-*XDH* ^*Tg*/ *Glau*^ = 3; *Mut*-*XDH* ^*Tg/Glau*^ = 3.

### Genomic sequencing

We performed whole-genome sequencing to identify the insertion-sites of transgene in the mouse genome. Paired-end 150 bp-long sequencing reads were aligned to a modified version of the reference genome (GRCm38/mm10) using the bowtie aligner (bowtie-bio.sourceforge.net). The reference was modified to include a copy of the original cloning vector. Sequencing reads that uniquely mapped both to the mouse genome and to the cloning vector sequence were used to localize the insertion sites. Raw-sequencing reads in the form of.fastq files were deposited in the European Nucleotide Archive with the accession (E-MTAB-5502). To generate the library, DNA was extracted from liver using the DNA Mini kit (Qiagen) and fragmented using Bioruptor (Diagenode). Briefly, 380 ng DNA in 100 microL in 500 microL tubes were subjected to sonication with the following parameters: power setting low, cycle 30 sec on/90 sec off, time 8 minutes in ice-cold, degassed water, resulting in average 550 bp size fragments. The library was prepared using the TruSeq Nano DNA kit (Illumina). DNA and library quantifications were performed with the Quant-iT PicoGreen dsDNA Kit (Invitrogen). Library quality was assessed by using the High sensitivity Bioanalyzer kit (Agilent).

### Western blot analysis

Western blot analyses were performed according to standard procedures^26^ using antibodies recognizing the following mouse proteins: anti-AP2β (NBP1-89063, Novus Biologicals Europe, Abingdon, UK); anti-AP2δ (NBP2-13428, Novus Biologicals Europe); anti-XDH^40^; anti-α-tubulin (T5168, Sigma Aldrich).

### Real Time PCR

To detect the transcripts encoding AP2β and AP2δ, we used specific Taqman assays: Tfap2b (Mm00493468_m1, Applied Biosystems); Tfap2d (Mm00462520_m1, Applied Biosystems). To measure the levels of the XDH mRNA we performed Sybr-green PCR assays using the following amplimers: 5′-AGAAAAATGCAGACCCTGAAACA-3′ (forward primer); 5′-CCGCACAGCCCCAACTT-3′ (reverse primer). The Taqman and Sybr-green assays were performed on total RNA extracted from eyes or liver according to standard procedures (Terao et al., 2016). For the XDH experiments, we used 4 animals/each experimental group, while for the AP2β and AP2δ studies we used 7 animals/each experimental group. To determine the CNV (Copy Number Variation) of the AK029562 non-coding gene we used a custom-designed Taqman assay (ID: AILJKZC, Life Technologies).

### Immunohistochemistry

Eyes from WT and transgenic animals were isolated and fixed in Davidson’s fixative. The whole globe was routinely processed for histology and properly oriented at the time of paraffin embedding. Microtomic sections (4–5 micrometers) were obtained from paraffin blocks and stained with hematoxylin and eosin or the Periodic acid–Schiff procedure (PAS) for histological examination. Serial sections were immunostained with the same anti-AP2β and anti-AP2δ antibodies as described in the previous section (diluted 1:300), using a standard avidin–biotin– peroxidase complex (ABC) method^41^. Antigen retrieval was performed by heating slides for 30 minutes at 95 °C in a water bath, in a citrate buffer solution (pH 6,5). 3-Amino-9-ethylcarbazole (AEC) was used as a chromogen and slides were then counterstained with Mayer hematoxylin and mounted with glycerin jelly. Negative controls were prepared by replacing the primary antibody with normal rabbit serum. Immuno-labelling of AP-2β and AP-2δ was quantitatively assessed using an automatic image analysis system (Image Pro Plus 4.5, Media Cybernetics): the total number of positively stained retinal cells in 5 high power fields (400x) was calculated. For these experiments we used 4 distinct animals/experimental group.

## Electronic supplementary material

Supplementary Figures
