# Supplementary material for: Generation of a new mouse model of glaucoma characterized by reduced expression of the AP-2β and AP-2δ proteins
Source: Sci Rep. 2017 Sep 11;7:11140. doi: 10.1038/s41598-017-11752-6 (PMC5593953; doi:10.1038/s41598-017-11752-6)
Supplement: Supplementary file 1 — Supplementary Figures [file 41598_2017_11752_MOESM1_ESM.pdf]

SUPPLEMENTARY INFORMATION

Title: Generation of a new mouse model of glaucoma characterized by reduced expression of the AP-2β and AP-2δ proteins

Maria Monica Barzago[1], Mami Kurosaki[1], Maddalena Fratelli[1], Marco Bolis[1], Chiara Giudice[2], Laura Nordio[2], Elisa Cerri[3], Luciano Domenici[3,4], Mineko Terao[1] and Enrico Garattini[1]

[1] Laboratory of Molecular Biology, IRCCS-Istituto di Ricerche Farmacologiche "Mario Negri", via La Masa 19, 20156, Milano, Italy.

[2] DIVET, Faculty of Veterinary Medicine, University of Milan, Italy. Via Celoria 10, 20113 Milano, Italy

SUPPLEMENTARY FIGURES

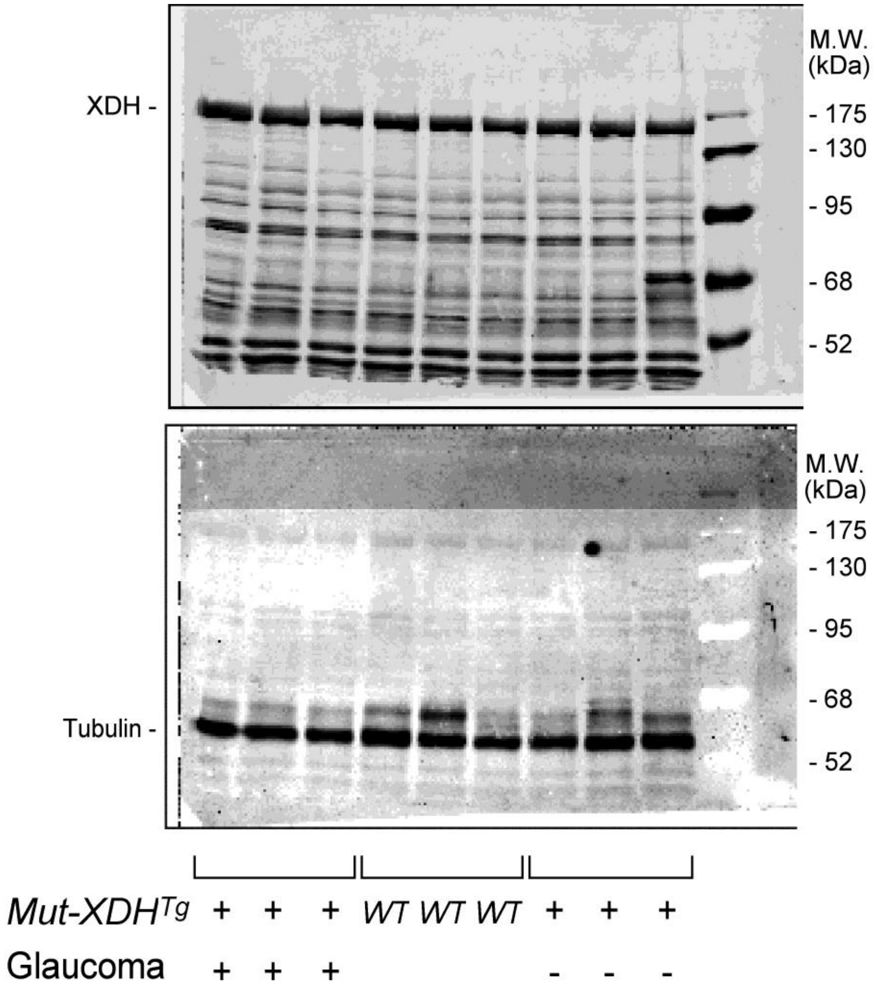

Fig. S1 Full-length blots/gels of Figure 4

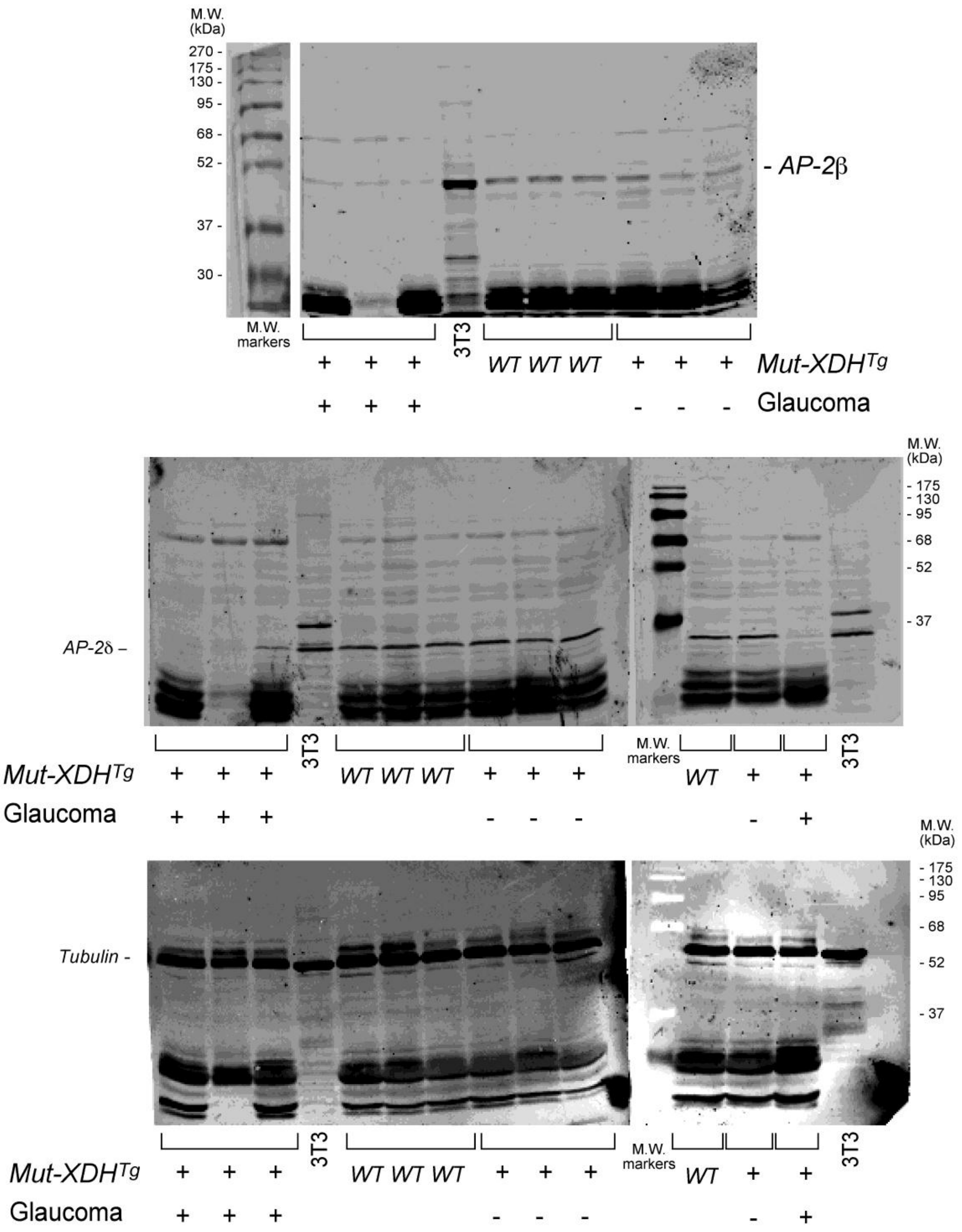

Fig. S2 Full-length blots/gels of Figure 6
